# Effect of welding parameters on microstructure and mechanical properties of dissimilar AISI 304/ductile cast iron fusion welded joints

**DOI:** 10.1038/s41598-024-70050-0

**Published:** 2024-08-27

**Authors:** M. El-Shennawy, H. A. Abdel-Aleem, M. M. Ghanem, A. M. Sehsah

**Affiliations:** 1 Mechanical Engineering Department, Faculty of Engineering, Helwan University, Helwan, Egypt; 2 Manufacturing Technology Department, Welding Technology & NDT Lab., Central Metallurgical R&D Institute (CMRDI), Cairo, Egypt

**Keywords:** AISI 304 stainless steel, Ductile cast iron (DCI), SMAW, Dissimilar welding, Microstructure characteristics, Mechanical properties, Engineering, Materials science

## Abstract

Problems associated with dissimilar fusion welding are mainly originated from the differences in melting points, coefficients of thermal conductivity and thermal expansion, …etc., and carbon content when welding dissimilar ferrous materials. In this study, the problems associated with dissimilar fusion welding of stainless steel AISI304 with ductile cast iron DCI grade A536 were investigated. Using shielded metal arc welding (SMAW) process, various welding parameters were studied to investigate the successful/accepted dissimilar welded joint(s). Welding electrodes and welding techniques were the main studied parameters. Microstructural and mechanical investigations were carried out for welded joints under different welding parameters. Tensile, impact and hardness tests coupled with optical and scanning electron microscopic examinations with EDX analysis were made for metallurgical and mechanical evaluations of welded joints. This extensive study could solve the problem of dissimilar welding between ductile cast iron and 304 stainless steel. The main results showed that joints welded by ENiCrFe-3 electrode in root pass and ENiFe-CI in filling passes were the successful dissimilar welded joints with 422 MPa tensile strength which represents 104% of annealed DCI base metal and without any changes in toughness properties, where toughness at HAZ of DCI was 18 J. High Ni content in weld metal increased the strength, ductility and reduced the weld metal dilution.

## Introduction

Joining of dissimilar metals in the industrial sector is required in various applications due to the need to combine different characteristics of the two joined metals which could give more advantages in service^1,2^. In particular, the dissimilar welding joints of ductile cast iron to stainless steel have attracted much attention due to cooperative advantages such as high strength, corrosion resistance, wear resistance and lower cost of ductile cast iron and favourable mechanical properties of stainless steel. It has been extensively applied in gas pipeline, pressure piping, petrochemical industry and power generation^3^. The challenge is to avoid the expected defects of such welded joint due to the difference in physical, chemical and thermomechanical properties between stainless steel and ductile cast iron. For instance, thermal conductivity, coefficient of thermal expansion and melting temperature are significantly different as well as the carbon migration in the weld zone^4^. According to high percentage of carbon in DCI 4.8% which diffuses into austenite during welding, that forms hard brittle phases, namely martensite and carbides at the interface^4^. Besides, the chemical composition of ductile cast iron, mechanical properties and structure^5–7^ play a great role in these joint defects. Preheat of DCI before welding can reduce cooling rate and decrease martensite formation in heat affected zone^8^. Preheating temperature range of ductile cast iron depends on the hardenability of the iron (chemical composition or carbon equivalent)^9^. Preheat temperature of DCI within the range 200–300 °C is adequate to decrease martensite formation in the heat affected zone^10^. Radoslawet et. al. have studied the dissimilar joint of stainless steel to ductile cast iron made by friction welding. Their observations showed more diffusion of carbon from base ductile cast iron to stainless steel and form chrome carbides at grain boundary^11^. Those findings are expected to be more extensive with fusion welding. The Ni-base consumable are extensively used for dissimilar metal welding joint fabrication because of certain advantages of carbon migration^12–14^. Higher nickel content can reduce the degree of carbon migration and martensite formation in the weld zone and its coefficient of thermal expansion is intermediate between dissimilar alloys^15^.

The buttering technique is a method used in dissimilar welding. This technique involves applying a layer of filler electrode to one side of the joint before welding, which stops the migration of carbon to weld metal and decreases the residual stresses in the welded joint^16^. Dinesh et al. have studied dissimilar welding of SA508Gr.3Cl.1 to AISI304 with and without buttering, they used Ni–Fe alloy for buttering a layer on one side of the joint and then filled v-groove with different electrodes; Inconel 82 (ERNiCr-3) and Inconel 182 (ENiCrFe-3). They obtained better mechanical properties with buttered joints compared to welded joints without buttering^17^. Same results were found by W. Winarto et al. who had studied the effect of buttering on dissimilar welded joint of low carbon steel to stainless steel^18^. Hamed safari et al. have studied the effect of buttering layer on dissimilar welded joint of API 5l X65 to AISI304. Buttering layer increased the heat input which decreased the strength of the welded joint, but the elongation was improved^19^.

El-Shennawy et al. have studied dissimilar welding of DCI to stainless steel using SMAW, their recommendations were showed that ENiFe-CI electrode is suitable for welding DCI to AISI304^20^. A. M. Sehsah et al. reported that Inconel 182 is suitable for weld ductile cast iron to stainless steel, high nickel content delayed the carbon diffusion to cast iron. In addition, the super alloy Inconel 182 have elements such as Cr, Ti and Nb which increased the strength of welded joint^21^. K. Devendranath et al. have studied dissimilar welding AISI304 and Monel 400 by GTA using different electrodes E309L, ENiCu-7 and ENiCrFe-3. They found that the mechanical properties of dissimilar welded joints using ENiCu-7 filler is better than E309L, as well as higher content of ENiCrFe-3 decreased the soft zone^22^. Super alloys Inconel 82^23^ and Inconel 182 have more applications in dissimilar welding^23,24^.

The problems associated with dissimilar welded joints between ductile cast iron and stainless steels in general and 304 grade in particular still exist in many applications. There are various interacting parameters controlling the quality of such joints which need to be extensively studied to declare the most appropriate welding parameters and welding conditions should be applied. This paper discusses the problems associated with dissimilar fusion welding of stainless steel AISI304 with ductile cast iron DCI grade A536. Extensive experimental studies using shielded metal arc welding (SMAW) process with various welding parameters were carried out to investigate the successful/accepted dissimilar welded joint(s). Welding electrodes and welding techniques were the main studied parameters. Microstructural and mechanical investigations were carried out for welded joints under different welding parameters.

## Experimental work

Dissimilar welded joints of ductile cast iron and stainless steel 304 were produced under various welding conditions using shielded metal arc welding process (SMAW) and tungsten inert gas (TIG). Welding electrode and welding procedure are parameters applied. Ductile cast iron was produced in a foundry at Casting Technology Department in Central Metallurgical R&D Institute (CMRDI). Chemical composition and mechanical properties of the cast were comparable to ASTM A536 grade [80-55-06] as depicted in Tables 1 and 2, respectively.

**Table 1:** Chemical composition of DCI and stainless steel 304.

Alloy	C	Mn	Si	Cr	Ni	Mo	Cu	P	S	Co	Mg	Fe
DCI (As cast)	3.83	0.28	2.66	0.038	0.27	0.007	0.278	0.022	0.012	0.0024	0.024	Rest
AISI 304	0.055	1.40	0.31	18.8	8.27	–	0.253	0.031	0.001	0.172	–	Rest

**Table 2:** Mechanical properties of DCI and stainless steel 304.

Alloy	Tensile strength, MPa	Yield Strength, Mpa	Elongation, %	Hardness, HB	Charpy Impact Toughness, J
DCI (annealed)	428	326	19	155	16
AISI 304	644	366	54	123	325

**Figure 1:**
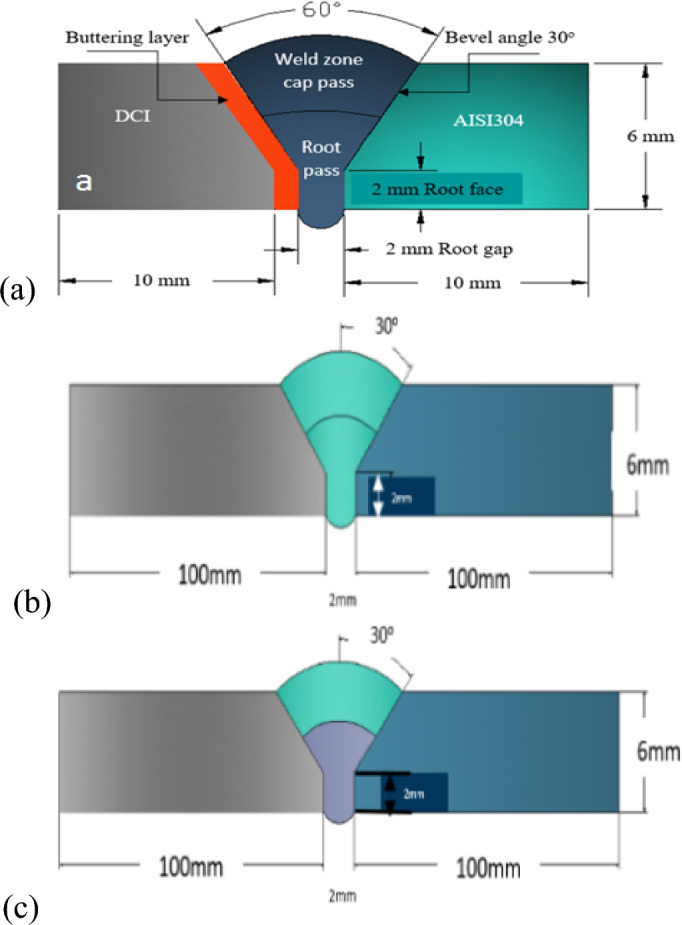
Weld joint configuration and techniques for welding AISI 304/DCI; (**a**) with buttering (**b**) filling with single electrode, (**c**) filling with multi electrodes for root and cap.

Produced DCI was annealed at temperature 900 ^○^C with soaking time 1.5 h. Annealing treats the segregation, improved ductility and elongation, decreased hardness and lowered the probability of finding cracks during welding. Joint configuration for all specimens used in this study is shown in Fig. 1. All Specimens were preheated to 250 °C and then welded by SMAW and TIG process. Welded specimens were isolated by thermal wool after welding process to avoid rapid cooling in heat affected zone of ductile cast iron. Welding electrodes used in this study included AL-bronze 90/10 (ER Cu Al-A2), Inconel 182 (ENiCrFe-3), cast-iron-Ni–Fe-electrode (ENiFe-CI) and E502-16. Welding procedures adopted for these dissimilar joints included using AL-bronze 90/10(ER Cu Al-A2) for butter DCI side and fill the joint using Inconel 182 (ENiCrFe-3), other sample welded using single type electrode for filling all groove root and cap, two types of electrodes one for root and the other for the cap V-groove. Welding conditions and procedures are detailed in Table. 3.

**Table 3:** welding condition and procedure.

Condition	Sample No	Pass	Electrode	Electrode diameter, mm	Type of welding	Ampere	voltage	Travel Speed mm/min	H.I. J/mm
With buttering	1	1^st^	ER Cu Al-A2 buttering	3	TIG	100	13	76.9	608.58
		2^nd^	ENiCrFe-3	3	SMAW	54	21	90.9	598.81
		3^rd^	ENiCrFe-3	3	SMAW	70	24	79.3	1016.89
Without buttering	2	1^st^	ENiFe-CI	3	SMAW	75	20	100	750
		2^nd^	ENiFe-CI	3	SMAW	75	20	104.71	687.61
	3	1^st^	ENiCrFe-3	3	SMAW	63	28	68.9	1228.91
		2^nd^	ENiFe-CI	3	SMAW	85	18	100	734.4
	4	1^st^	ENiCrFe-3	3	SMAW	54	28.3	133.3	550.28
		2^nd^	E502-16	3	SMAW	80	32.2	126.58	976.83

The buttering technique followed in this study was in the form of surfacing by deposition one layer of weld metal using AL-bronze 90/10(ER Cu Al-A2) electrode on the surface of DCI side. The buttering layer ranged from 2–3 mm thickness. This technique is used to provide a suitable transition weld deposit for subsequent completion of the butt weld on the groove face of one member which is DCI. Buttering here provides a layer that can act as a barrier between the two metals; DCI and AISI304 and prevent them from bonding together directly as they have different expansion rates.

Welded specimens have been examined visually, by dye penetrant test (PT) and x-ray test (RT) according to American Society of Mechanical Engineers (ASME) code Sect. 5^25^ to assure quality and the results were accepted. The welded samples were prepared for macro and microstructures investigations by grinding and polishing then etched using 2% Nital reagent for ductile cast iron side of welded joint flowed by electro-etching using oxalic acid for austenitic stainless-steel side of the dissimilar joint. Optical and electron microscope (SEM) equipped with energy dispersive spectroscopy (EDS) are used for microstructure examination and phase analysis of the welded specimen.

**Figure 2:**
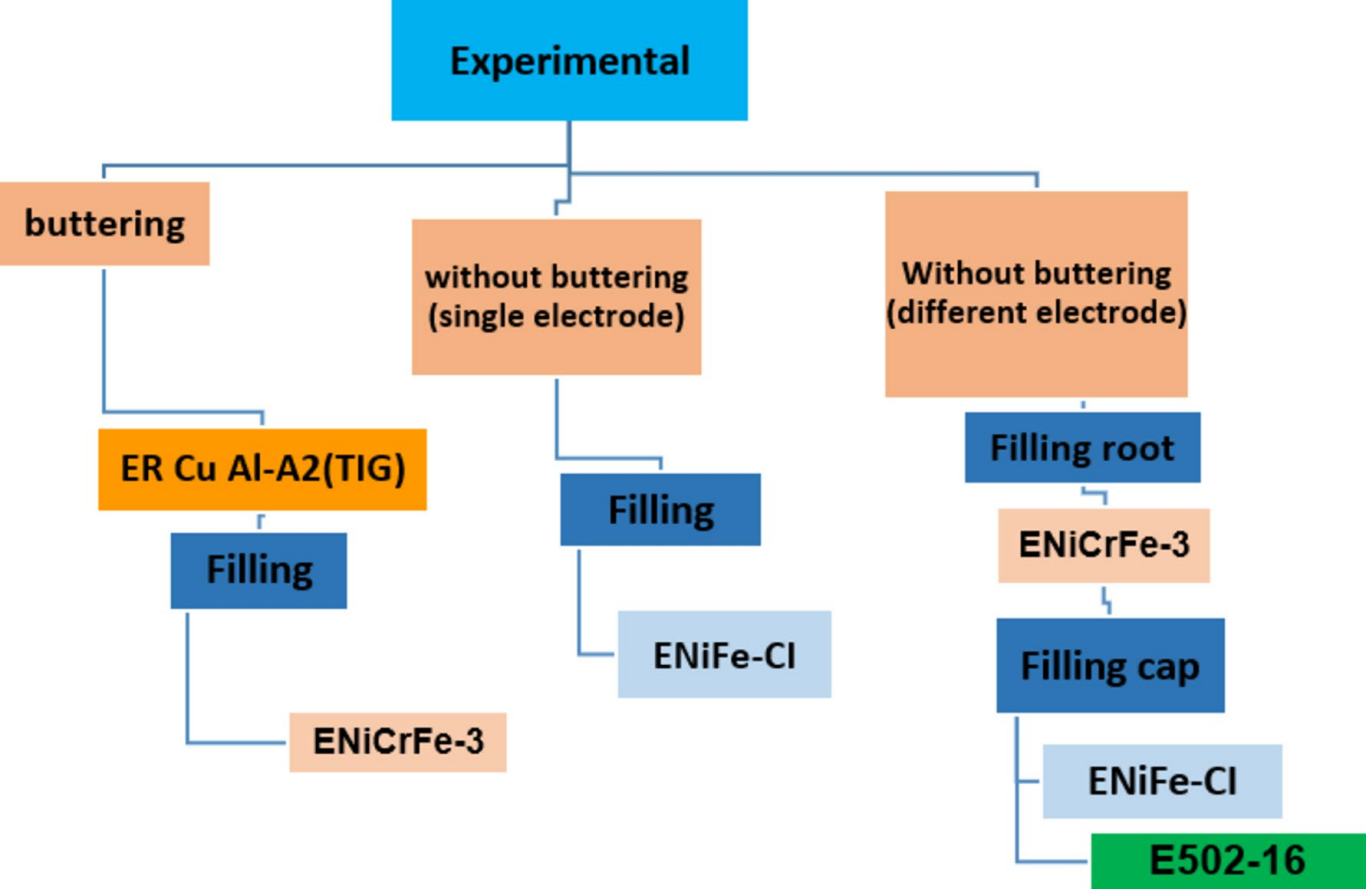
Flow chart for welding techniques/procedures followed in current research.

The main three techniques followed in this study to weld DCI/304 stainless steel plates are summarized in Fig. 2 which shows schematically, butter DCI side and filling use single electrode, use single and multi different electrodes for the same joint; in case of multi different electrodes one for root pass and other for filling passes. Flow chart for welding techniques/procedures followed in this research is shown in Fig. 2**.**

**Figure 3:**
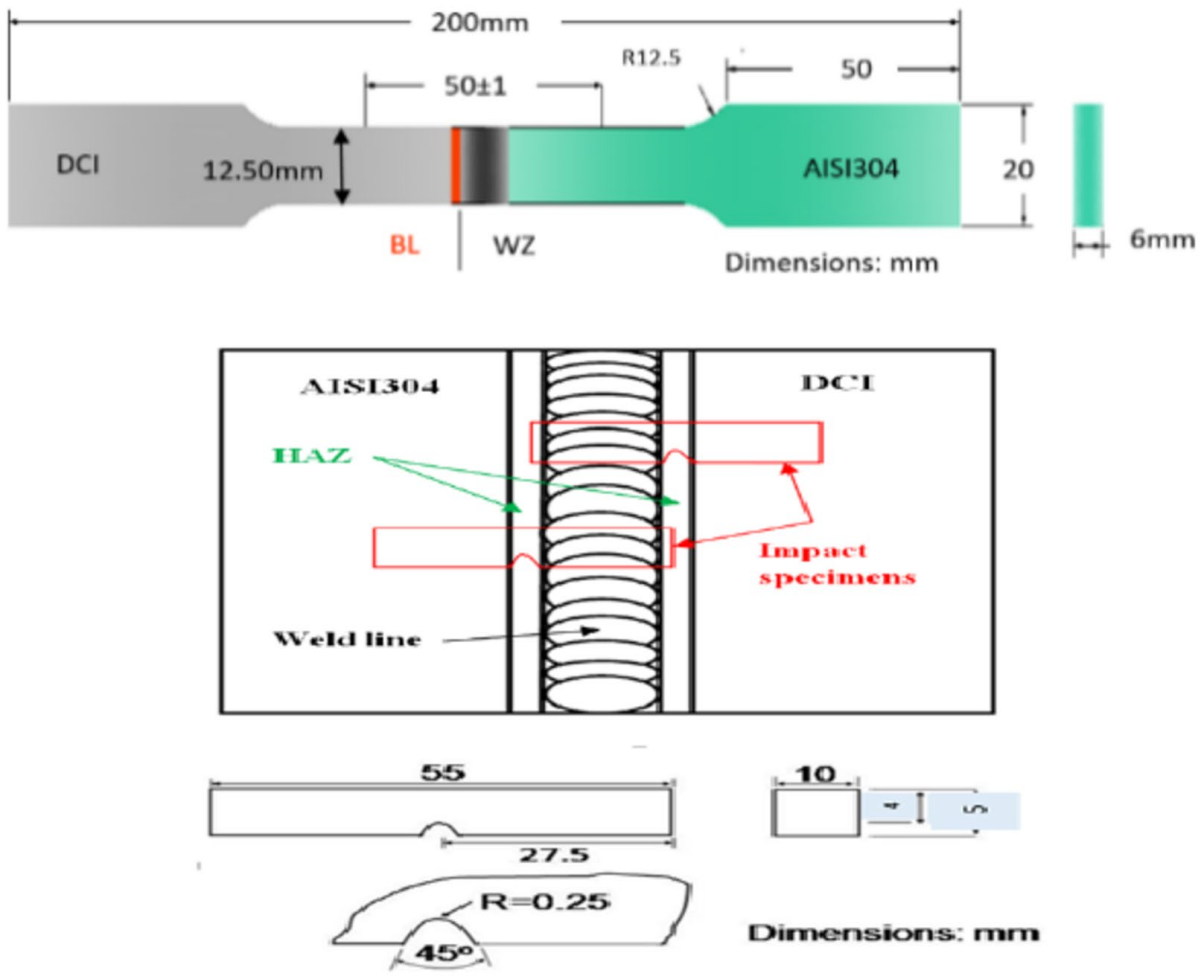
Schematic illustration showing locations shape and dimensions of the tensile and impact test specimen.

Vickers micro-hardness measurements are carried out across cross sections of welded joint samples. Tensile and impact test were carried out for welded specimens according to American Society of Mechanical Engineers (ASME) code section IX^26^, as shown in Fig. 3. Impact, fracture toughness test is carried out at room temperature on standard machined specimens taken from predetermined locations in HAZ of DCI and other specimens taken in HAZ of AISI304 welded joints according to ASME code section IX.

## Results and discussions

### Visual inspection

#### Macro-structure of dissimilar welded joints

**Figure 4:**
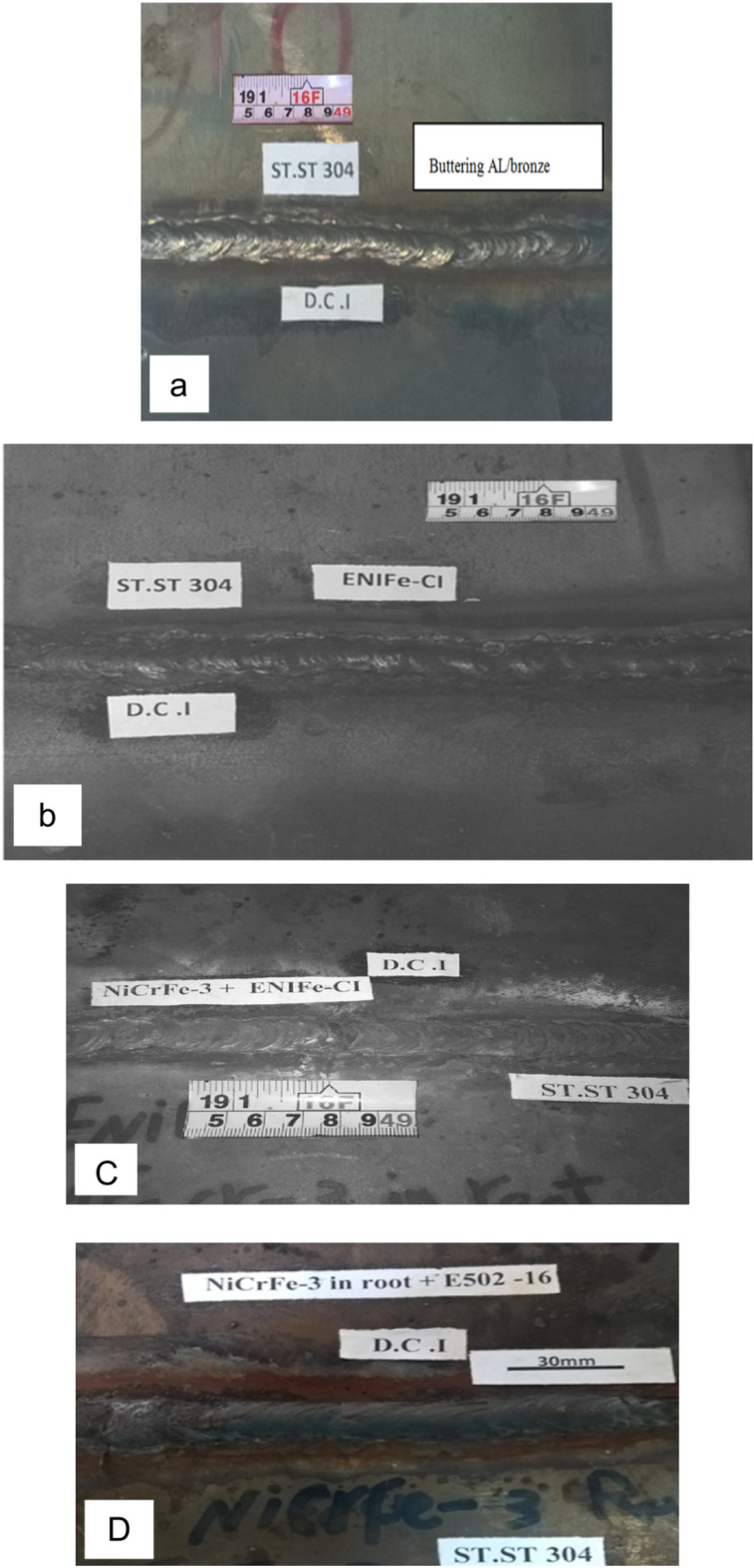
Photographs of dissimilar welded joints.

Examples of general and close-up views of as-welded joints are shown in Fig. 4. No visible welding defects are detected.

**Figure 5:**
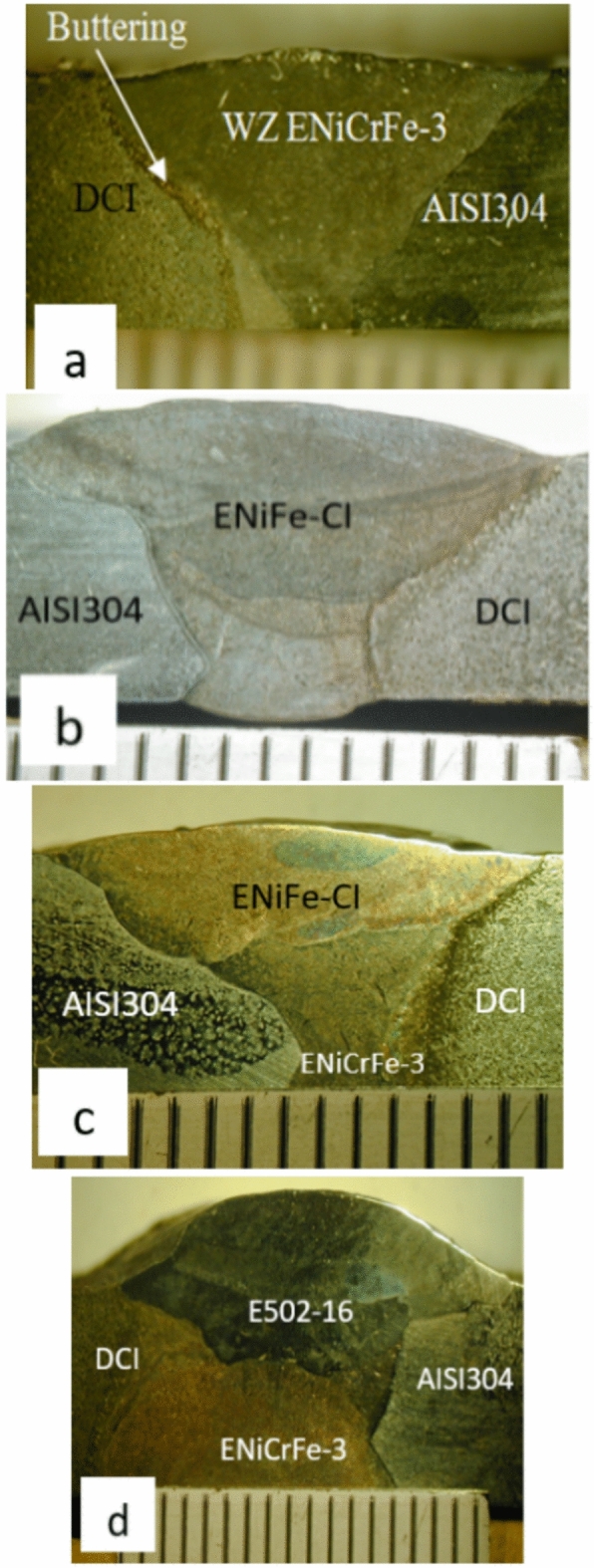
Photographs of cross section area of dissimilar welded joints, (**a**) butter DCI using AL-bronze &fill ENiCrFe-3, (**b**) filling V groove root and cap passes by ENiFe-CI, (**c**) filling root by ENiCrFe-3 and complete V groove passes by ENiFe-CI, (**d**) Filling root by ENiCrFe-3 and complete V groove passes byE502-16.

General and close up views of cross sections of welded joints are shown in Fig. 5 No welding defects are detected. Figure 5 shows that the curve of cap pass for three specimens are smooth and good root penetration as well as smooth weld bead reinforcement morphology is obtained.

### Microstructure of dissimilar welded joints

A **Effect of buttering technique on microstructure of welded joint**

**Figure 6:**
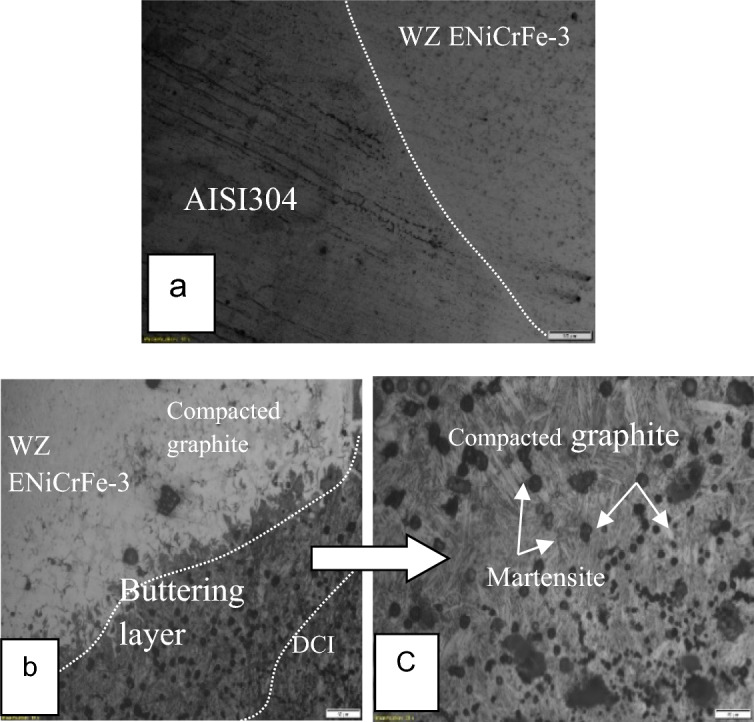
Microstructure of dissimilar welded joint using AL/bronze for buttering DCI side and filling the gap of the joint using ENiCrFe-3, (**a**) the interface at AISI304/WZ, (**b**) the interface at DCI/WZ, (**c**) Magnification of microstructure buttering layer.

Ductile cast iron edge was buttered using Al-bronze filler (ER Cu Al-A2) and the dissimilar joint welded using ENiCrFe-3 electrode for filling V-groove. The microstructure of sample 1 weld joint at the DCI/WZ interface is shown in Fig. 6 Microstructure of ductile cast iron base metal consists of graphite nodules formed in ferritic matrix. Compacted graphite and martensite in the buttered and HAZ zones are obvious. The weld zone (WZ) consists of lamellar grey and round white contrast phases formed in austenitic matrix. No visible graphite in WZ was detected.

**Figure 7:**
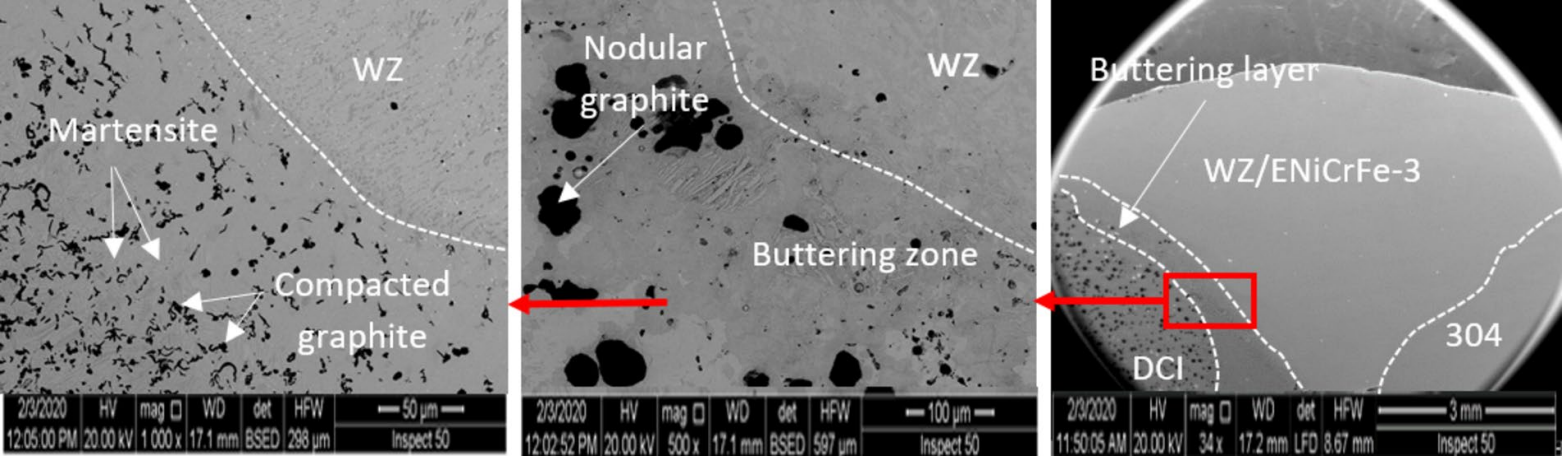
SEM micrographs of sample 2 welded joint at DCI/WZ interface are showing microstructure of ductile cast iron base having graphite nodules.

SEM microstructures of sample 1 weld joint at the DCI/WZ interface are shown in Fig. 7 Microstructure of ductile cast iron base metal consists of graphite nodules formed in ferritic matrix. Compacted graphite and martensite in the buttered and HAZ zones are obvious. The weld zone (WZ) consists of lamellar grey and round white contrast phases formed in austenitic matrix. No visible graphite in WZ was detected.

Results of EDX analysis of WZ matrix revealed existence elements of Ni–Fe–Cr–Mn–C. In addition, Cu–Al was detected in WZ due to dilution of the WZ with buttered layer of Cu-10Al filler. Al was detected in the buttered layer which might form Al-carbide, which highly expect to increase hardness in the buttered layer. It was also obvious that Cr dissolved in WZ matrix are reduced to 10 wt.% from original content of filler metal composition (17% Cr) due to formation of Cr-carbide and Nb-carbide precipitates phase. EDX analysis of the white round phase in WZ reveals that this phase is Nb-carbide.

Heat affected zone of DCI revealed lamellar phase, with EDX analysis of these phase it showed Si, Fe-carbides with composition. This phase is formed due to a rapid cooling. In buttering layer, the existence element revealed C–Al–Si–Cr–Fe–Ni–Cu–Nb which form Al, Nb, Cr, Fe– Carbides.

B **Effect of welding electrode type on microstructure of welded joints**

Different filler and electrode types ERCuAl-A2, ENiCrFe-3, ENiFe-CI and E502-16 which were used to study the effect of electrode type on microstructure and mechanical properties of dissimilar AISI304/DCI weld joints.

**Figure 8:**
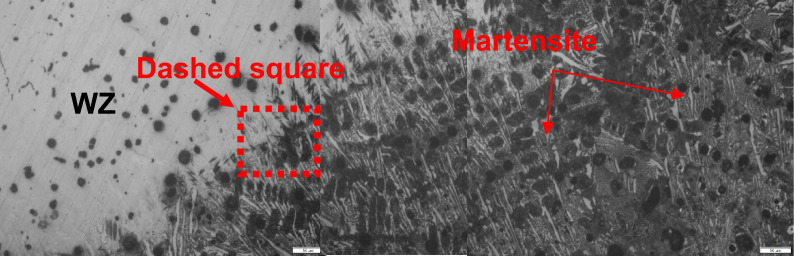
Microstructure at DCI/WZ interface of sample 2 welded using (ENiFe-CI) electrode.

**Figure 9:**
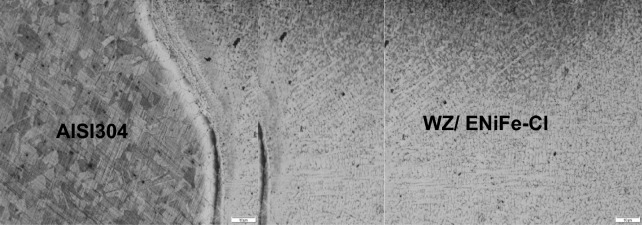
Microstructure at AISI 304/WZ interface of sample 2 welded using (ENiFe-CI) electrode.

**Figure 10:**
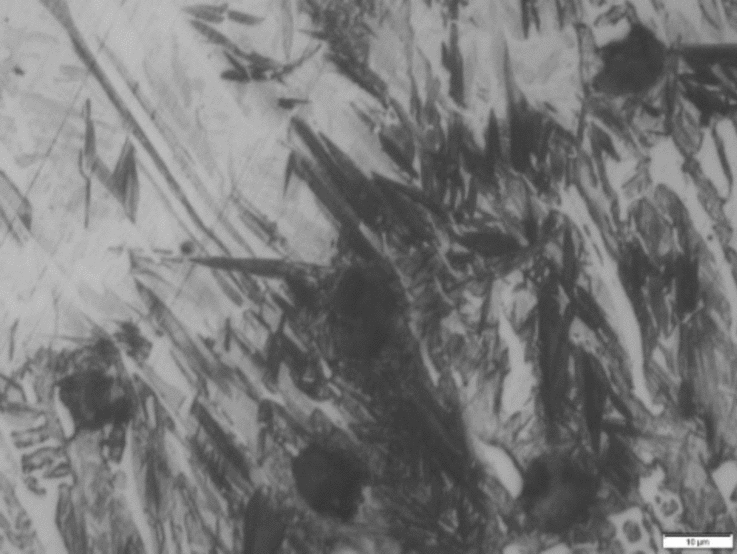
Microstructure of dashed area at DCI/WZ interface of sample 2 welded using (ENiFe-CI) electrode.

Microstructure of dissimilar weld joint for sample 2, using ENiFe-CI is shown in Fig. 8 and the microstructure at AISI304/WZ interface is shown in Fig. 9. This microstructure mainly consists of two phases at weld zone. Fine graphite black contrast phase formed in austenitic white contrast matrix. No carbides are visible in the matrix on using ENiFe-CI compared to ENiCrFe-3 electrode, Figs. 8 and 10. Some martensite and iron carbide are visible at DCI/WZ interface due to rapid solidification at weld interface compared with weld zone slower solidification rate as shown in Fig. 8. Figure 10 show the magnification of martensite formation at DCI/WZ interface at dashed square area (Fig. 9).

**Figure 11:**
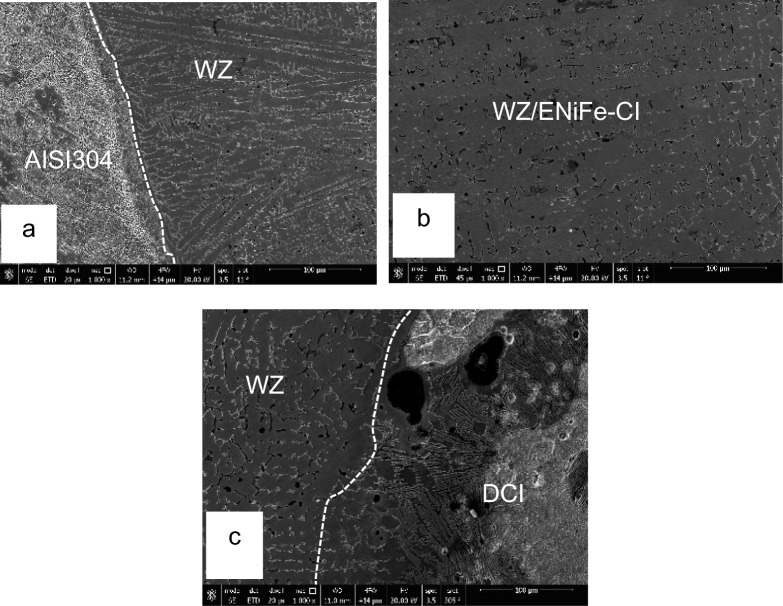
SEM microstructure of sample 2 joint welded using ENiCrFe-3.

SEM microstructure of sample 2 welded using ENiFe-CI shows the microstructure of ductile cast iron base metal consists of two phases with black contrast round phase (graphite) formed in ferritic matrix. Microstructure of weld zone reveals that microstructure mainly consists of two phases at weld zone. Fine graphite black contrast phase formed in austenitic white contrast matrix. No carbides are visible in the matrix when using ENiFe-CI compared to ENiCrFe-3 electrode, Fig. 11. Figure 11a shows the weld zone at the AISI304 side. Figure 11b shows the weld zone. Figure 11c shows the weld zone at the DCI side. Some martensite is visible at DCI/WZ interface due to rapid solidification at weld interface compared with weld zone slower solidification rate as shown in Fig. 11c.

**Figure 12:**
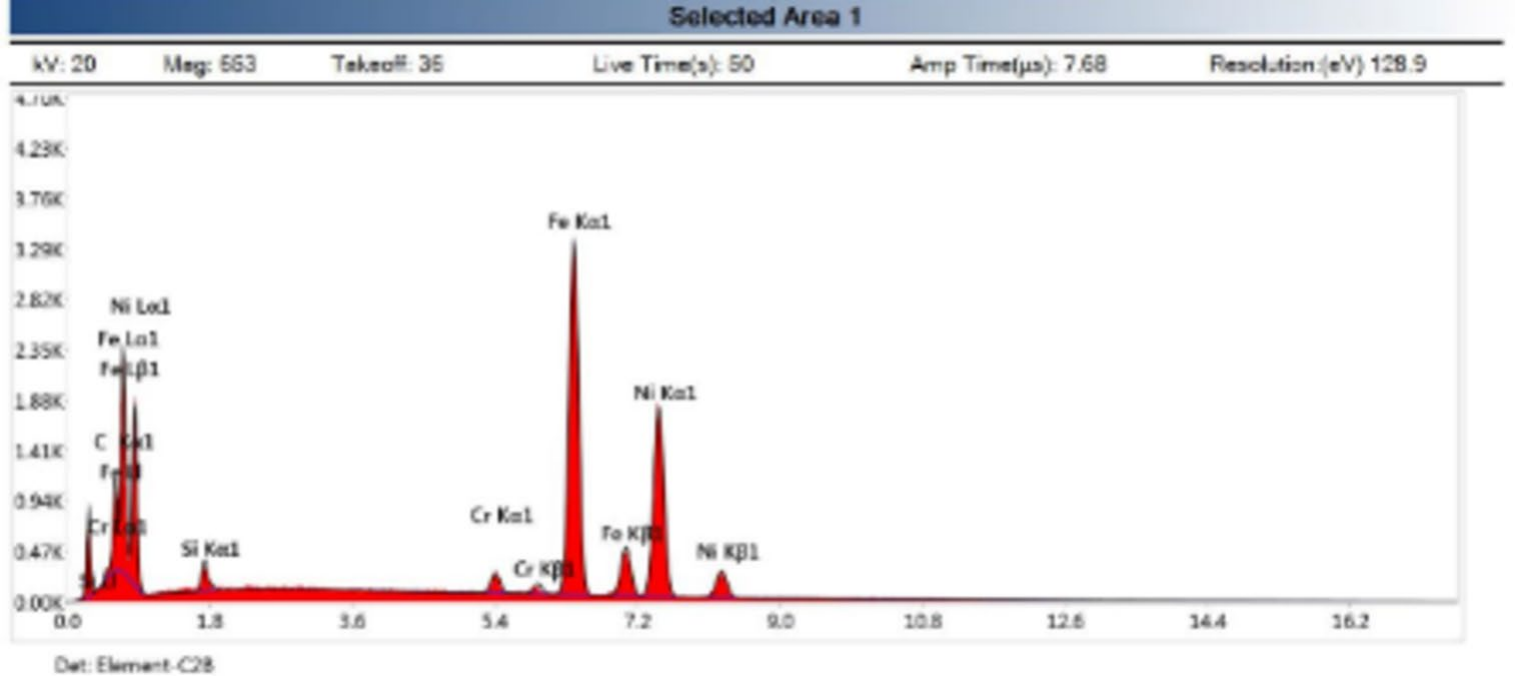
EDX analysis of weld zone revealed phases are C–Si–Cr–Fe and mainly Ni phase of sample 2.

**Figure 13:**
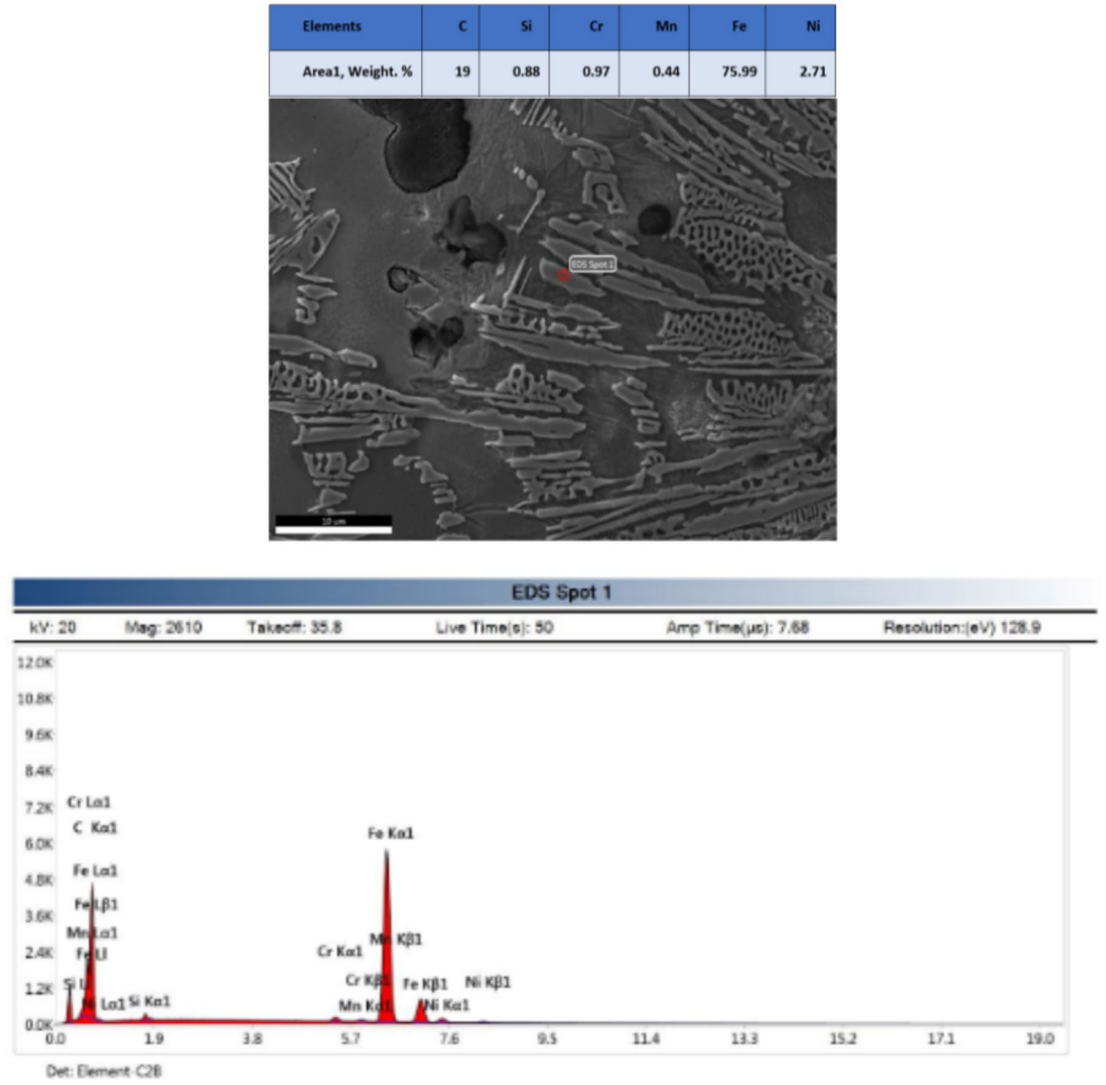
EDX analysis at DCI/WZ interface revealed iron carbide of sample 2.

EDX analysis at weld zone revealed phases which are C–Si–Cr–Fe and mainly Ni phase as shown in Fig. 12. EDX analysis at DCI/WZ interface revealed iron carbide phase as shown in Fig. 13.

**Figure 14:**
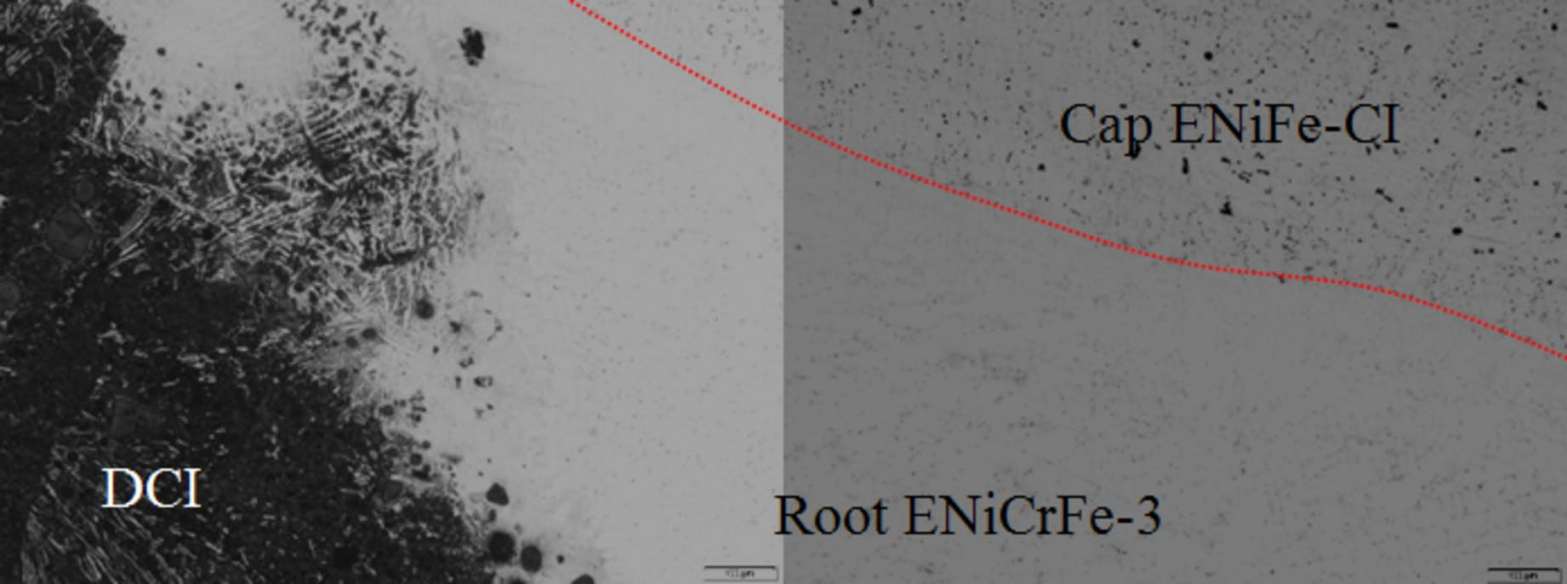
Microstructures of DCI/WZ interface of joint welded by combination ENiCrFe-3 & ENiFe-CI for specimen 3.

**Figure 15:**
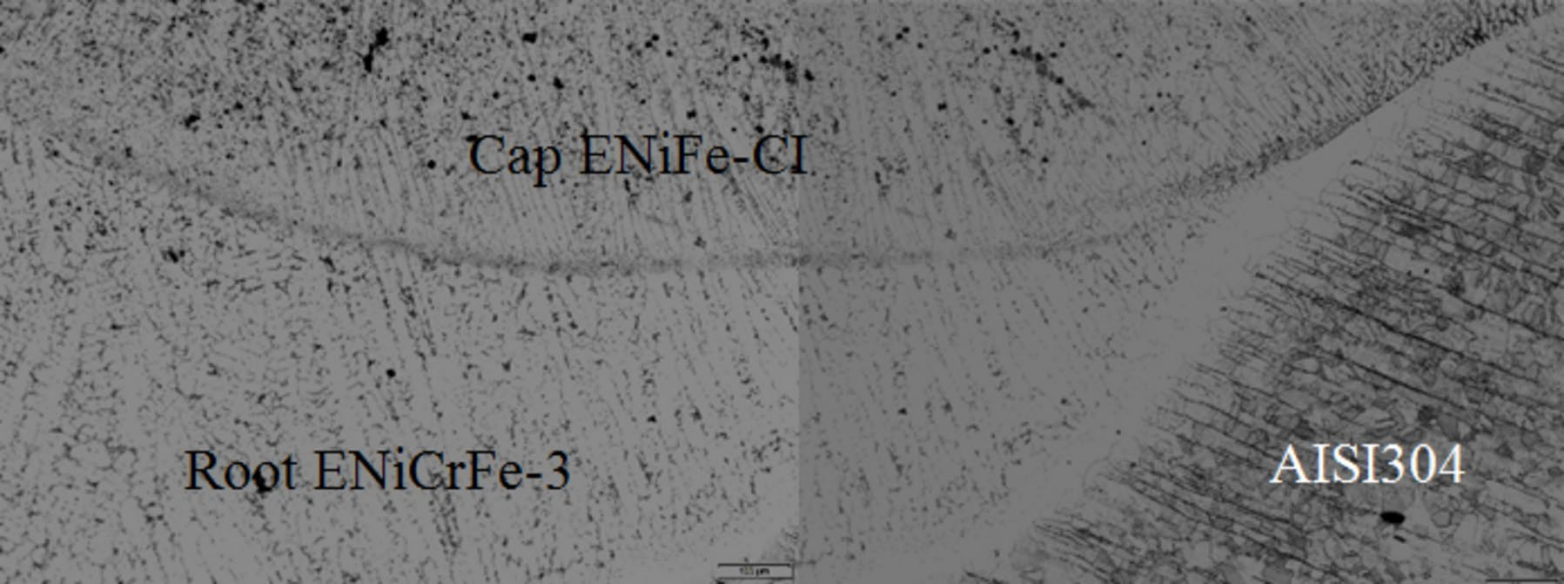
Microstructures of 304 stainless/WZ interface of joint welded by combination ENiCrFe-3 & ENiFe-CI for specimen 3.

Optical micrographs for sample 3 near DCI/WZ interface and 304 stainless/WZ interface are shown in Figs. 14 and 15, respectively. While, fine irregular graphite phase formed in WZ cap, no graphite phase formed at root pass. Fine irregular graphite phase formed at WZ cap is attributed to dilution of DCI base metal with ENiFe-CI. On using ENiCrFe-3 for root pass, no graphite was formed since carbon react with Cr, Nb and Ti to form Cr-carbide, (Nb, Ti) carbide instead of graphite formation, Figs. 14 and 15.

**Figure 16:**
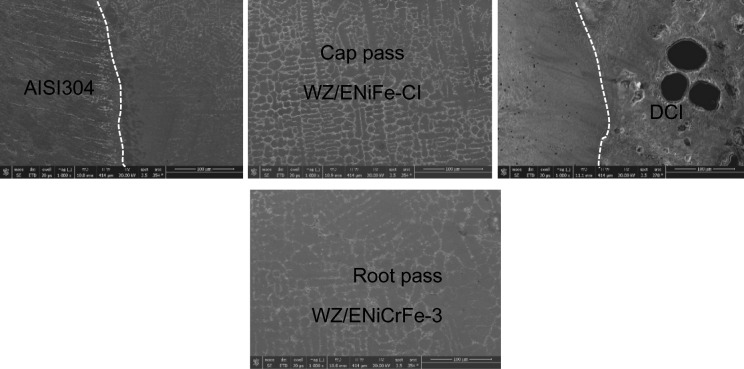
SEM micrographs of specimen 3 joint welded using ENiCrFe-3 and ENiFe-CI for root and cap passes, respectively.

SEM microstructure of specimen 3 joint welded using ENiCrFe-3 and ENiFe-CI for root and faces passes, respectively is shown in Fig. 16. Microstructure of ductile cast iron base metal consists of two phases with black contrast round phase (graphite) formed in ferritic matrix. Microstructure of weld zone root pass consists mainly of three phases white round contrast phase, white irregular phase formed in grey matrix. Weld zone microstructure at face pass reveals that microstructure mainly consists of three phases black contrast having irregular and round morphology and white round phases formed in grey phase matrix.

EDX analysis results of phases exist in sample joint 3 together with their corresponding peaks. EDX analysis at AISI304/WZ face interface phases revealed that the irregular black contrast phase mainly consists of C–Cr–Fe in the form of carbides.

EDX analysis of the weld zone cap phases revealed black matrix phase mainly consists of Fe, Ni in addition to irregular and round morphology phase mainly consists of C, Cr, Fe, Ni and a white contrast phase at root pass mainly (Nb, Ti) carbide precipitate and formation of Cr-carbides.

**Figure 17:**
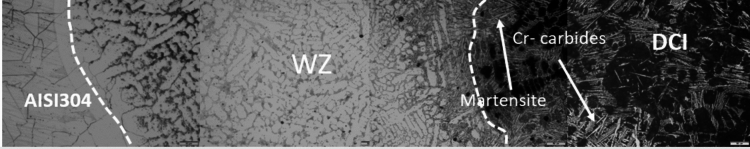
Microstructures of sample 4 welded using ENiCrFe-3 and E502-16 for root and cap respectively.

Microstructures of sample 4 welded using two electrodes namely; ENiCrFe-3 and E502-16 for root and face respectively are shown in Fig. 17.

At the DCI side of the dissimilar joint, microstructure at the partial fusion zone (PFZ) near root pass welded using ENiCrFe-3 electrode mainly consists of graphite nodules (black contrast) and Cr-Carbide lathe formed from the melt (bright contrast). Moving towards weld zone (WZ), microstructure mainly consists of fine secondary Cr-carbide (grey contrast) phase precipitated in austenitic matrix during solid state phase transformation.

Microstructures of sample 4 at AISI 304 side near interface between root and face passes is shown in Fig. 17. At 304 stainless side, PFZ root pass welded using ENiCrFe-3 electrode consists of grey Cr-carbide phase with irregular shape formed in austenitic matrix. On moving towards centre of weld zone (WZ), austenitic equiaxed grains are coarse compared with columnar grains at the PFZ.

**Figure 18:**
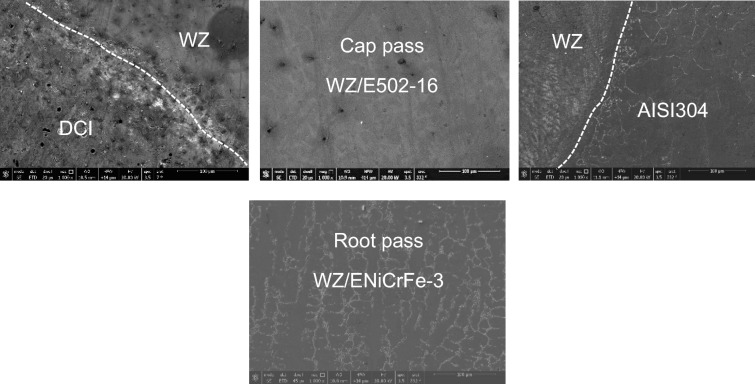
SEM micrographs of sample 4 joint welded using ENiCrFe-3 and E502-16 for root and cap passes, respectively.

SEM Microstructures of sample 4 welded using two electrodes namely, ENiCrFe-3 and E502-16 for root and face respectively are shown in Fig. 18. At the DCI side of the dissimilar joint, SEM microstructure at the partial fusion zone (PFZ) near root pass welded using ENiCrFe-3 electrode mainly consists of graphite nodules (black contrast) and Cr-Carbide lath formed from the melt (bright contrast). Moving towards weld zone (WZ), microstructure mainly consists of fine secondary Cr-carbide (grey contrast) phase precipitated in austenitic matrix during solid state phase transformation.

At the AISI 304 side, SEM microstructure show grey Cr-carbide phase with irregular shape formed in austenitic matrix is shown in Fig. 18.

EDX analysis of phases exist at DCI/WZ face interface of sample 4 together with their chemical composition revealed white round contrast phase which is mainly (Nb) carbide precipitate and formation of Cr-carbides lath. EDX analysis of phases exist in weld zone cap pass sample joint 4 revealed exist phases is mainly consisting of C–Cr–Fe in form Cr-carbides.

### Microhardness evaluation of welded joints

**Figure 19:**
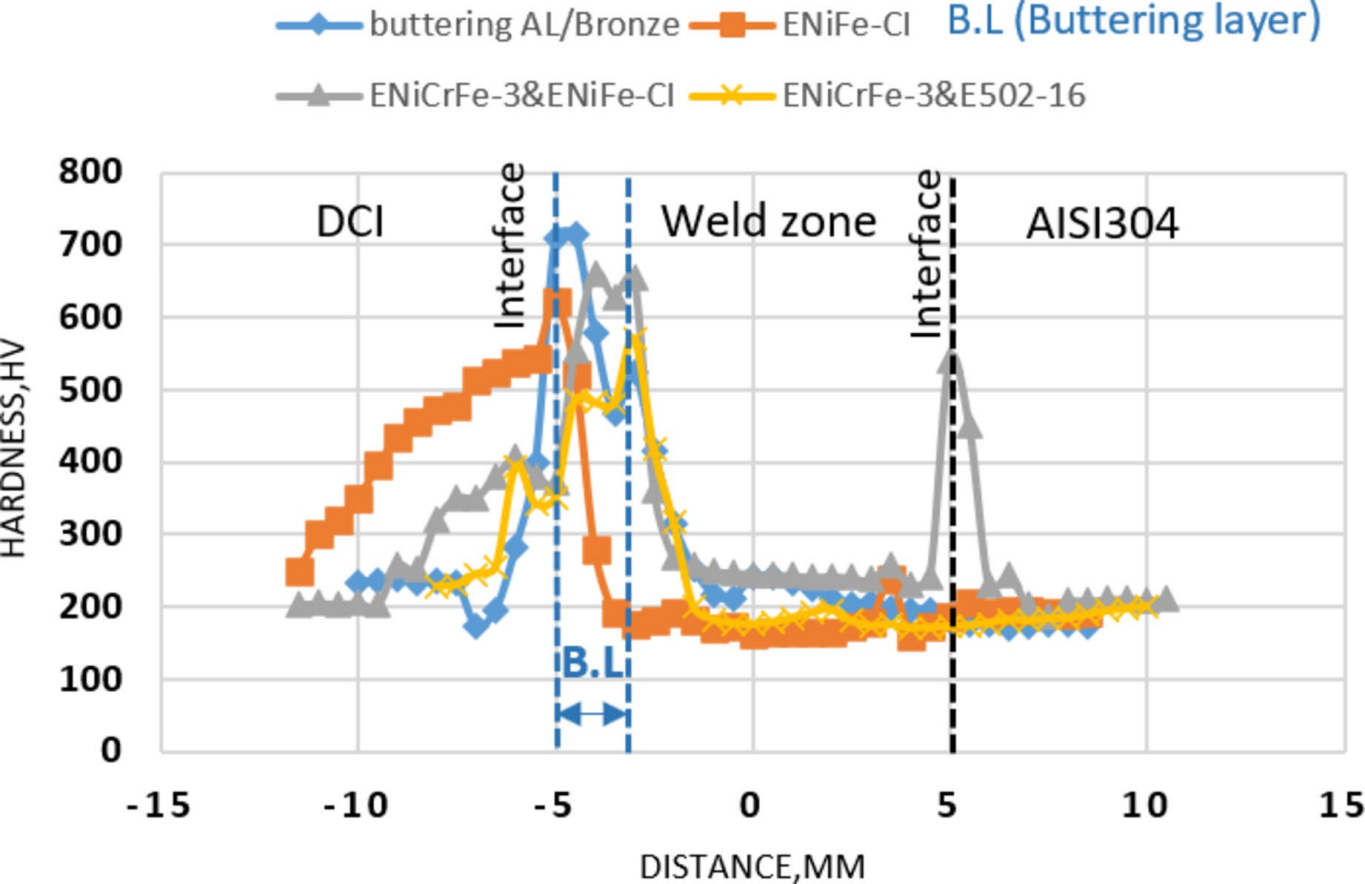
Change in hardness from the weld centreline of dissimilar welded joint.

When using AL-bronze filler for buttering DCI side, the hardness increased at HAZ due to martensite formation. The increase in hardness at DCI/WZ interface is attributed to formation of aluminium carbide. Aluminium in filler metal reacts with carbon to form aluminium carbide; the reason why hardness drastically increased at fusion line is shown in Fig. 19 and gives hardness 700HV. However, existence of aluminium in the butter layer prevents carbon migration from DCI side to the weld zone.

Figure 19 shows the change in hardness from the centreline of dissimilar welded joint sample 2 welded using ENiFe-CI electrode, the hardness in HAZ of DCI is recorded (450)HV but in PFZ give hardness (560) HV as a result of rapid cooling and formation martensite structure, while the hardness in WZ which have dendrite structure was (180–240) HV, but the hardness at WZ/AISI304 interface did not exceed 185HV. The microstructure of base AISI304 shows ferrite pearlite and recorded hardness of (140–170) HV as shown in Fig. 19, the hardness of DCI was in the range of (255–320) HV.

In sample 3 hardness at DCI/WZ recorded (500–580) HV; at root/cap interface in weld zone the hardness was (175–92) HV as shown Fig. 19. HAZ consists of martensitic structure and gives hardness of (480) HV, at AISI304/WZ interface the hardness recorded was (200) HV.

Figure 19 shows the change in hardness from the weld centreline of dissimilar welded sample 4 welded using two electrodes ENiCrFe-3& E502-16 for root/face, respectively. The hardness profile location at root/face interface, the hardness at DCI/WZ interface reaches to (650) HV according to Cr-carbide formation in PFZ. Heat affected zone consists of needle martensite structure which increased the hardness to (400–500) HV.

### Tensile test (TS)

The tensile specimens are cut by electron discharge machine (EDM) (wire cutting machine), the specimens are perpendicular to the weld bead according to standard tensile specimens and procedure as per American Society of Mechanical Engineers (ASME) code section IX. Flat tensile specimens having 50 mm gage length, 6 mm thickness, 12.5 mm width (in the gage length region), 20 mm width of grip section and overall length of 200 mm, according to ASME code section. The tension was carried out on Shimadzu 1000 kN universal testing machine, the process is performed at a constant crosshead displacement rate 8 mm/min at room temperature.

**Figure 20:**
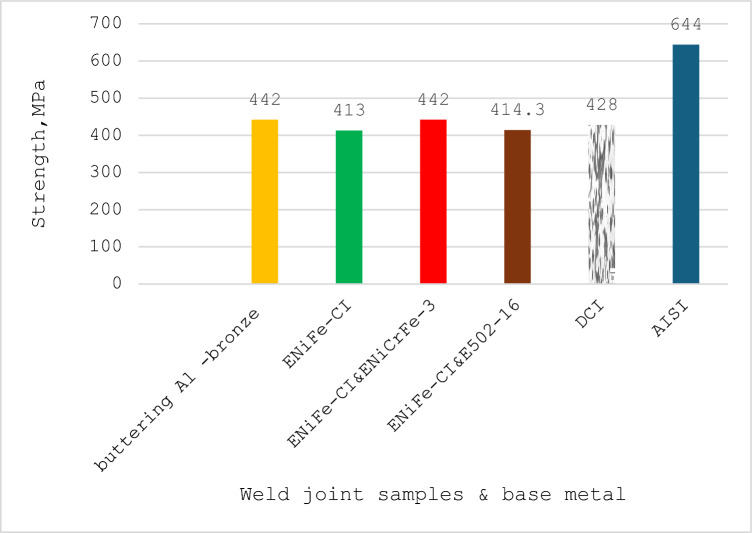
Tensile strength for welded joints with various electrodes.

Figure 20 shows the tensile strength results for welded joints. In general, the results of tensile test are correspondent with the results of hardness test. Most of samples failed at PFZ such as samples 1, 3 and 4 but sample 2 failed at weld zone.

Sample No. 1 having buttered with ERCuAl-A2 and filled with ENiCrFe-3 electrode had the same tensile strength (TS) of 422 MPa of sample 3 which was welded using ENiCrFe-3 & ENiFe-CI for root and cap respectively. This value (422 MPa) of tensile strength is higher than that of base ductile cast iron and fracture location was in partial fusion zone (PFZ) of DCI side. Tensile strength of samples 2 welded using ENiFe-CI showed same value of 414 MPa for that of sample 4 which was welded using ENiCrFe-3 & E502-16 for root and cap respectively. Sample 2 failed at weld zone while sample 4 failed at PFZ of DCI side.

### Tensile test specimen fractography

**Figure 21:**
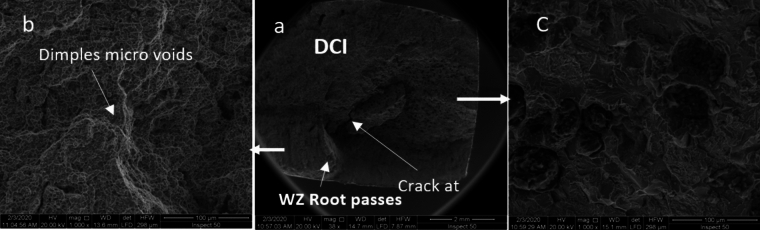
SEM Fractography of tensile tested sample 1 showing (**a**) general view of the weld joint fracture surface, (**b**) ductile fracture weld zone and (**c**) mixed ductile and brittle fracture of ductile iron. Cracks are visible at the ductile iron/weld zone interface.

**Figure 22:**
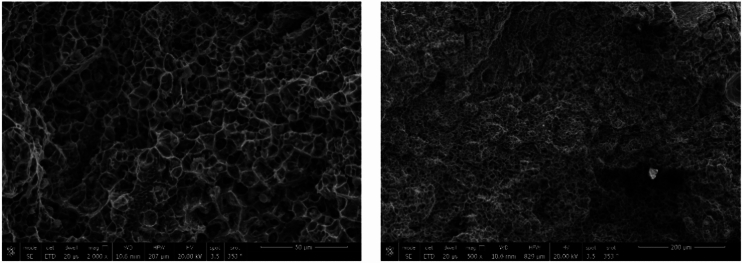
SEM fractography of tensile tested sample 2 at different magnification.

**Figure 23:**
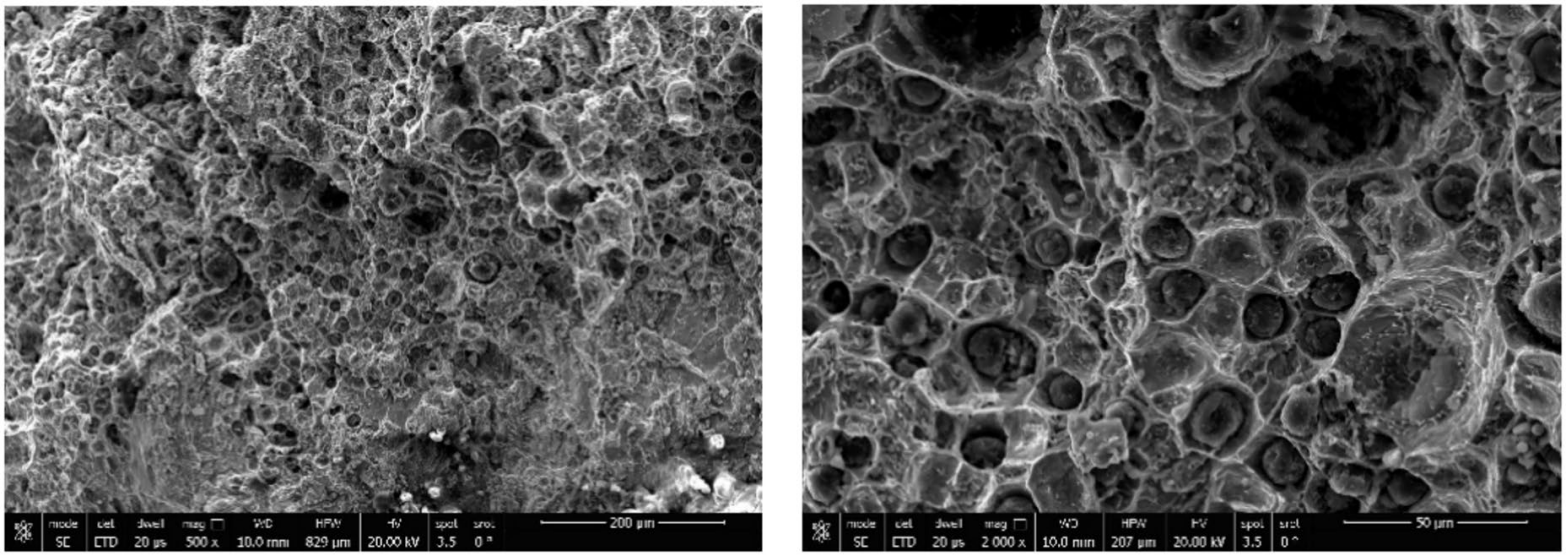
SEM fractography of tensile tested sample 3 at different magnification.

**Figure 24:**
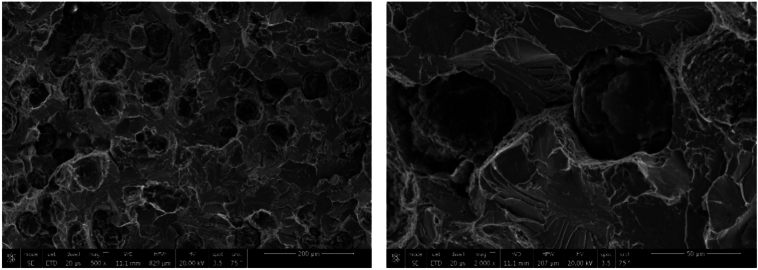
SEM fractography of tensile tested sample 4 at different magnification.

SEM fractography of sample 1 revealed ductile fracture of weld zone (WZ) when using ENiCrFe-3 welding electrode while mixed ductile and brittle fracture of ductile cast iron, and no graphite can be seen in WZ as shown in Fig. 21. A crack is observed at ductile cast iron/weld zone interface due to the highest hardness value at this zone. SEM fractography of sample 2 revealed ductile fracture of weld zone (WZ) when using ENiFe-CI welding electrode, Fig. 22. No graphite can be seen in weld zone. SEM fractography of tensile tested specimen 3 revealed brittle fracture at DCI/WZ interface when using ENiCrFe-3 and ENiFe-CI for root and cap passes, respectively, Fig. 23. SEM fractography of tensile tested sample 4 revealed brittle fracture at DCI/WZ interface on using ENiCrFe-3 and E502-16 for root and cap passes, respectively, Fig. 24.

### Impact test

**Figure 25:**
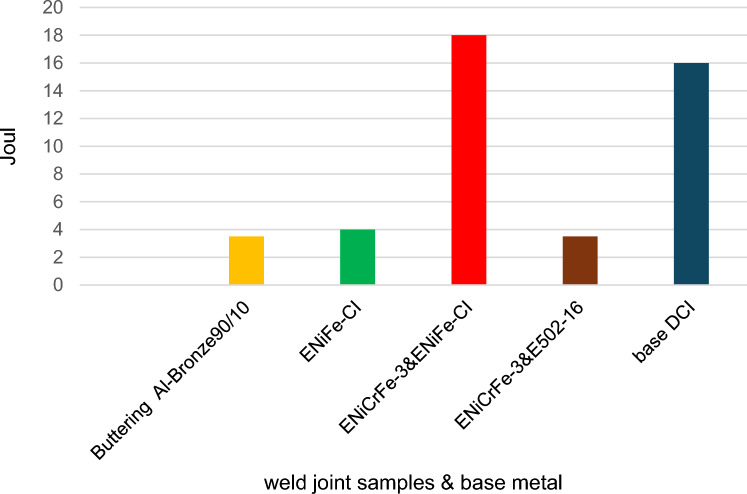
Bar graph of impact toughness results in HAZ of DCI side.

**Figure 26:**
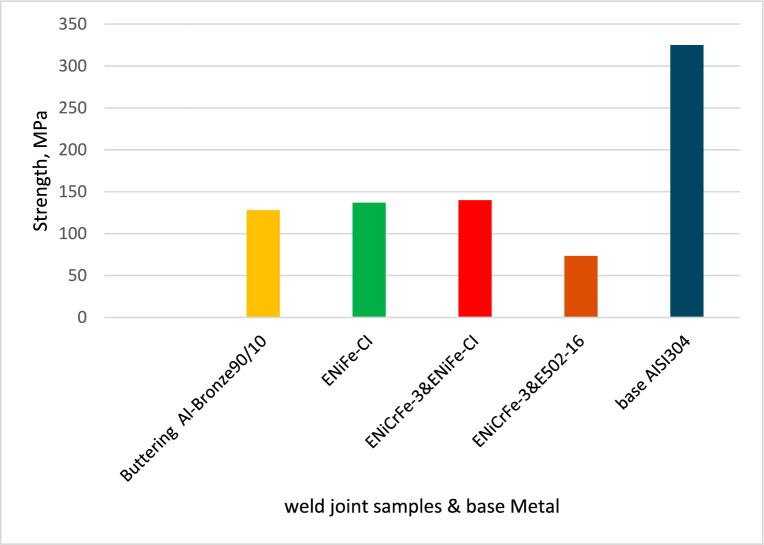
Bar graph of impact toughness results in HAZ of AISI304 side.

Impact test was carried out at room temperature on standard machined specimens taken from predetermined locations in HAZ of DCI while the other specimens taken in HAZ of AISI304 welded joints according to ASME code section IX. The impact values are so close to each other except sample 3 welded using ENiCrFe-3 and ENiFe-CI for root and cap respectively which showed impact toughness 18J. This attributed to high elongation of ENiCrFe-3 electrode, the impact values at DCI side are lower compared to AISI304 side due to high concentration of carbon content at HAZ near DCI as show in Figs. 25 and 26.

**Figure 27:**
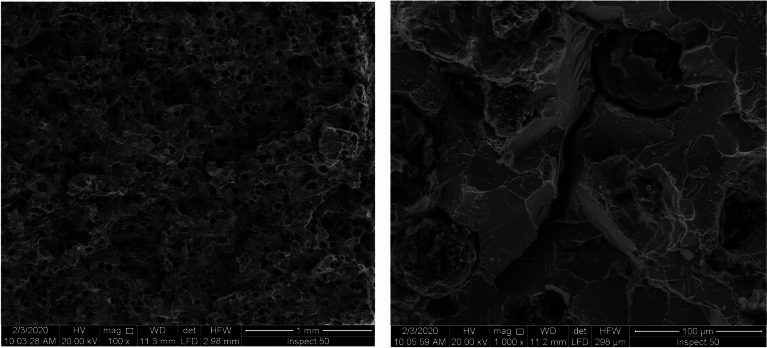
SEM fractography of impact tested specimen 3 at different magnifications.

SEM fractography of specimen 3 is shown in Fig. 27. Typical brittle fracture of the ductile cast iron is visible, and the fracture initiated at graphite nodules and propagating through the ferritic matrix. Misfit between graphite nodules and matrix is obvious and could be attributed to the difference in crystal structure of ferritic matrix and graphite.

## Conclusions

This study discusses the problems associated with dissimilar fusion welding of stainless steel AISI304 with ductile cast iron DCI grade A536. Using shielded metal arc welding (SMAW) process, various welding parameters were studied to investigate the successful/accepted dissimilar welded joint(s). Welding electrodes and welding techniques (use of different buttering layers) were the main studied parameters. Microstructural and mechanical investigations were carried out for welded joints under different welding parameters. This extensive study could solve the problem of dissimilar welding between ductile cast iron and 304 stainless steel. The following conclusions can be drawn from this study:

1. Using filler electrode (ERCuAl-A2) for buttering DCI side and filling the V-groove with electrode (ENiCrFe-3) gives high strength and hardness due to formation Al, Cr, Nb carbides at DCI side due to dilution of buttering layer with weld zone. It is worth mentioning that when using buttering technique at DCI side, it acts as obstacle layer to minimize carbon migration from DCI side to the weld and reduces formation of carbides in weld metal.
2. Using ENiCrFe-3 for root pass gives good root penetration due to high percent of Ni 59% as well as the content elements such as Nb, Ti and Cr which improves strength, hardness and corrosion resistance in addition to complete the filling cap passes with ENiFe-CI to avoid the excessive formation of Cr-carbides which make the joint very hard.
3. Using E502-16 electrode for filling cap instead of ENiFe-CI increases the hardness at weld zone interface.
4. Therefore, when welding dissimilar joint of stainless steel AISI304 with ductile cast iron DCI grade A536 it is recommended to use ENiCrFe-3 for root pass and complete filling passes with ENiFe-CI. This will give good penetration with good strength and toughness with decreasing chromium carbide formation.

This filed of dissimilar welding/joining still needs a lot of investigations to fulfil the increasing needs to minimize the weight and cost of products through joining dissimilar materials. This is a promising future work.

## Data Availability

Data is provided within the manuscript. All data generated or analyzed during this study are included in this manuscript.
